# Healthcare practitioner perceptions on barriers impacting cannabis prescribing practices

**DOI:** 10.1186/s12906-022-03716-9

**Published:** 2022-09-08

**Authors:** Yasmina Hachem, Sara J. Abdallah, Sergio Rueda, Jessica L. Wiese, Kamna Mehra, Jennifer Rup, Juthaporn Cowan, Antonio Vigano, Cecilia T. Costiniuk

**Affiliations:** 1 Medical Cannabis Program in Oncology, Cedars Cancer Center, McGill University Health Centre, 1001 boulevard Decarie, Montreal, QC H3A 3J1 Canada; 2 Division of Infectious Diseases, Department of Medicine, The Ottawa Hospital and Ottawa Hospital Research Institute, 501 Smyth Road, Ottawa, ON K1H 8L6 Canada; 3 Centre for Addiction and Mental Health, Institute for Mental Health Policy Research, 33 Ursula Franklin St, Toronto, ON M5S 2S1 Canada; 4 Campbell Family Mental Health Research Institute, Centre for Addiction and Mental Health, 250 College Street, Toronto, ON M5T 1R8 Canada; 5 Department of Psychiatry, University of Toronto, 250 College Street, Toronto, ON M5T 1R8 Canada; 6 Institute of Medical Science, University of Toronto, 1 King’s College Circle, Toronto, ON M5S 1A1 Canada; 7 Division of Infectious Diseases and Chronic Viral Illnesses Service, McGill University Health Centre, Infectious Diseases and Immunity in Global Health, Research Institute of McGill University Health Centre, McGill Research Center for Cannabis, McGill University, Montreal, Canada

**Keywords:** Medical cannabis, Attitude of health personnel, Canada, Covid-19, Survey

## Abstract

**Background:**

Canadians seeking medical cannabis (MC) may encounter difficulties in finding a healthcare provider (HCP) who authorizes their access to it. Barriers that HCPs face in authorizing MC are unclear. The objectives of this study were to evaluate HCP opinions, knowledge, comfort, and practice in MC prescribing and counseling on recreational cannabis use, and whether the COVID-19 pandemic affected MC prescribing practices.

**Methods:**

Eligible participants included HCPs (e.g., attending physicians, nurses, pharmacists) in Canada. A questionnaire evaluating their knowledge, comfort, and practice in medical and recreational cannabis was designed based on instruments developed in previous studies. Between April 13^th^-December 13^th^ 2021, ninety-one healthcare associations were asked to distribute the survey to their members, and an advertisement was placed in the online Canadian Medical Association Journal. Descriptive statistics were used to analyze the results.

**Results:**

Twenty-four organizations agreed to disseminate the survey and 70 individuals completed it. Of respondents, 71% were attending physicians or medical residents, while the remainder were nurses, pharmacists or other HCPs. Almost none (6%) received training in MC in professional school but 60% did receive other training (e.g., workshops, conferences). Over half (57%) received more questions regarding MC since recreational cannabis was legalized, and 82% reported having patients who use MC. However, 56% felt uncomfortable or ambivalent regarding their knowledge of MC, and 27% were unfamiliar with the requirements for obtaining MC in Canada. The most common symptoms for recommending MC were pain and nausea, whereas the most common conditions for recommending it were cancer and intractable pain. The strongest barrier to authorizing MC was uncertainty in safe and effective dosage and routes of administration. The strongest barrier to recommending or authorizing MC was the lack of research evidence demonstrating its safety and efficacy. During the pandemic, many respondents reported that a greater number of their patients used cannabis to relieve anxiety and depression.

**Conclusions:**

Our results suggest that HCPs across Canada who responded to our survey are unfamiliar with topics related to MC. The strongest barriers appear to be lack of clinical research, and uncertainty in safe and effective MC administration. Increasing research, training, and knowledge may help HCPs feel more equipped to make informed treatment/prescribing decisions, which may help to improve access to MC.

**Supplementary Information:**

The online version contains supplementary material available at 10.1186/s12906-022-03716-9.

## Background

In recent years, the legal landscape of medical and recreational cannabis has witnessed dramatic changes in Canada. In 2001, the *Marihuana Medical Access Regulations (MMAR)* established guidelines for Canadians to obtain legal authorization to produce their own cannabis plant, designate someone to produce for them, or to purchase from Health Canada’s supply [1]. In 2014, the MMAR was replaced by the *Marihuana for Medical Purposes Regulations (MMPR)*, which allowed individuals legal authorization to access dried cannabis produced by commercial licensed producers [1]. The MMPR was subsequently replaced by the *Access to Cannabis for Medical Purposes Regulations (ACMPR)* in 2016 [1], which sets out provisions for individuals to (i) produce a limited amount of cannabis for their own purposes and (ii) purchase dried cannabis, cannabis oil or starting materials from licensed producers [1, 2]. As outlined in the ACMPR, individuals seeking cannabis for medical purposes (CMP) are required to submit a medical document to a commercial licensed producer [2]. This authorization to purchase CMP must be obtained from designated health care practitioners (HCPs): physicians or nurse practitioners.

The laws governing access to medical cannabis (MC) under the ACMPR have not changed since the nationwide legalization of cannabis on 17 October, 2018, under *The Cannabis Act *[2]. Thus, despite the legalization of recreational cannabis, individuals seeking CMP are still required to obtain authorization from an HCP. Individuals registered as CMP users benefit from: HCP assessment of indications, contraindications, and limitations to CMP, professional oversight on both the treatment plan and its adjustments over the course of follow-up, and financial relief (e.g., MC can be claimed as a medical expense under Revenue Canada’s Medical Expense Tax credits and registered MC users have access to compassionate pricing from most commercial licensed producers).

Canadians seeking MC encounter difficulties, however, in finding HCPs who support their application to access it [3], even since the introduction of *The Cannabis Act* and legalization of recreational cannabis. One study examining barriers to access found that Canadians faced difficulty finding preferred products legally; other barriers were cost and difficulties using the legal access system, including difficulty obtaining authorization [4]. Another study found that Canadians often encountered resistance from their neurologists and existing care teams when seeking MC authorization for their children’s drug-resistant epilepsy [5]. In America and Australia, HCPs cite lack of knowledge of cannabinoids and uncertainty regarding good prescribing^1^ practices as barriers to supporting CMP [6–10]. Barriers that Canadian HCPs face in supporting applications for MC under the ACMPR are not clear [11]. It is therefore important to understand HCP knowledge of and comfort level in CMP in order to inform policies that will improve safe and effective access. Furthermore, given the broad impact of the COVID-19 pandemic on many aspects of human health, understanding how the pandemic may have influenced HCPs’ practices is important. The objectives of this study were to evaluate (i) HCP knowledge of, comfort in, and practice of cannabis for medical and recreational purposes, (ii) HCP opinions on various topics related to cannabis for medical and recreational purposes, and (iii) how the COVID-19 pandemic may have affected their cannabis prescribing practices.

## Methods

### Survey design

An anonymous survey was electronically administered to HCPs in English or French to evaluate HCPs’ knowledge of, comfort in, and practice of medical and recreational cannabis, using REDCap to capture survey results. This questionnaire was based on instruments developed in previous studies[9, 10, 12] and can be found in the Supplementary Materials section. Part I of the survey collected demographic information (e.g. age, sex, role, years in practice, geographic location). Part II evaluated participant knowledge, comfort and practice of CMP. Participants were presented with four statements and asked to select one that best reflected their current prescribing practices. The statements were: “I do not recommend or prescribe medical cannabis, nor do I refer patients to a person/clinic with expertise in medical cannabis for further evaluation” (Group A); “I do not recommend or prescribe medical cannabis, but I refer patients to a person/clinic with expertise in medical cannabis for further evaluation” (Group B); “I recommend medical cannabis and refer patients to a person/clinic with expertise in medical cannabis for further evaluation as I do not prescribe it” (Group C); “I recommend and prescribe medical cannabis if I think it is appropriate without referring patients to a person/clinic with expertise in medical cannabis” (Group D). Based on this selection, participants completed different subsets of questions related to their knowledge of MC and barriers to prescribing and/or recommending it. If participants did not make a selection here (Group E), they were asked questions on various topics related to MC, including research priorities and their preferred methods of learning. Parts III and IV of the survey were presented to all participants and collected information on practice, knowledge and comfort in recreational cannabis use as well as the impact of the COVID-19 pandemic on cannabis practice. The survey included four parts and used a combination of Yes/No, checkbox, and Likert scale questions. A Likert scale is a popular tool used to measure attitudes, knowledge, perceptions, values, and behavioral modifications. Such a scale involves a series of statements that respondents may choose from in order to rate their responses to evaluative questions [13]. For example, respondents were asked to rate several potential barriers to recommending or prescribing MC, from 0 to 5, where a rating of 0 indicated “no barrier at all” and a rating of 5 indicated “very large barrier”.

### Recruitment

Eligible participants included HCPs (e.g., attending physicians, nurse practitioners, physician assistants, pharmacists) working in Canada. We contacted 91 professional and national organizations (Supplementary Table 1) and requested that our survey be disseminated on their website, or directly to their members via e-mail. An e-advertisement was also published in the online Canadian Medical Association Journal weekly news. Study data was collected and managed by the Data Management Centre of the Centre for Outcomes Research and Evaluation (CORE) at the Research Institute of the McGill University Health Centre (RI-MUHC). The survey was open from April 13^th^, 2021- December 13^th^, 2021. Descriptive statistics were used to summarize responses using IBM SPSS Statistics 25. Ethics approval was obtained from the MUHC REB (2021–7584).

## Results

Of the organizations contacted, 24 (26%) agreed to circulate the survey either to all members, or to members within their institutions only (Supplementary Table 1). It is unclear how many may have circulated it without confirming with us first, and how many potential respondents received the survey after distribution. This makes a true response rate not possible to calculate, especially considering that many mailing lists and association memberships overlap.

**Fig. 1:**
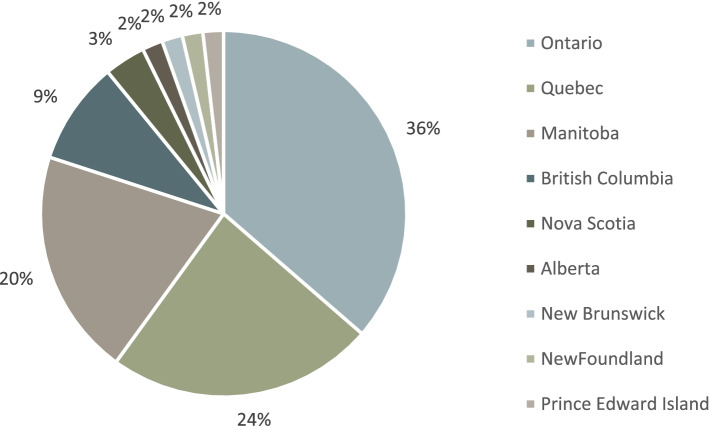
Survey respondents’ province of clinical practice

A total of 117 participants accessed the survey. Forty-seven participants were excluded from analysis. Fourteen participants were excluded for completing or partially completing Part I of the survey (demographics) but providing no other survey responses, and 33 participants accessed the survey but provided no responses. Participants were not excluded if they skipped some demographic questions but completed the survey otherwise. A final sample of 70 HCPs were included in the analysis. For the purposes of analysis, Groups A and B were combined, as both groups neither recommended nor prescribed MC. Of the final sample, 16 (23%) neither recommend nor prescribed MC (Group A + B); 14 (20%) recommend MC when indicated but refer patients to a person/clinic with expertise in MC for prescription (Group C); 25 (36%) recommend and prescribe MC themselves (Group D). Fifteen participants (21%) chose not to answer the question (Group E). Survey respondent demographics by group are shown in Table 1 and Fig. 1.

**Table 1:** Survey respondent demographics

	Group A + B N (%) or Mean ± SD	Group C N (%) or Mean ± SD	Group D N (%) or Mean ± SD	Group E N (%) or Mean ± SD
Number of Respondents	16 (23%) A = 5, B = 11	14 (20%)	25 (36%)	15 (21%)
Age in years	50.9 ± 13.1	44.7 ± 11.8 ^*^	55.0 ± 10.8	50.4 ± 13.5
Years of practice	20.7 ± 12.7	15.4 ± 12.3 ^*^	26.0 ± 12.8	21.7 ± 12.6 ^#^
Sex				
Male	4 (25%)	6 (43%)	14 (56%)	11 (73%)
Female	11 (69%)	8 (57%)	11 (44%)	4 (27%)
Prefer not to say	1 (6%)			
Born in Canada	12 (75%)	11 (79%)	16 (64%)	14 (93%)
Role				
Attending physician	8 (50%)	8 (57%)	24 (96%)	8 (53%)
Registered nurse	5 (31%)	2 (14%)		3 (20%)
Pharmacist	2 (13%)	3 (21%)		1 (7%)
Other	1 (6%)	1 (7%) – resident physician	1 (4%) – family physician at a cannabis clinic	3 (20%)

^*^ *n* = 13

^#^ *n* = 14. *SD* standard deviation

Of all respondents, 71% were attending physicians or residents, while the remainder were nurses (*n* = 10, 14%), pharmacists (*n* = 6, 9%) or other HCPs (*n* = 1, 1%). The respondents were mostly (*n* = 42, 60%) from Ontario and Quebec and had been in practice for a mean of 21.85 (± 12.92) years. Over 94% of all respondents did not receive any training in cannabinoid-based medicines (CBM) in professional school but 10% did receive practical training outside of medical school or residency programs, and 53% received other training (e.g., workshops, conferences); Table 2 depicts a descriptive analysis of training and educational activity undertaken by respondents in relation to MC.

**Table 2:** Medical cannabis training and educational activity of survey respondents

	**Group A + B *n* = 16**	**Group C *n* = 14**	**Group D *n* = 25**	**Group E *n* = 21**
**Training in School**	0 (0%)	3 (21%)	1 (4%)	0 (0%)
< 4 h		3		
> 15 h			1	
**Practical Training**	1 (6%)	2 (14%)	2 (8%)	2 (13%)
< 4 h	1	1		1
5–9 h		1	1	
> 15 h			1	1
**Other Training**	5 (31%)	10 (71%)	18 (72%)	4 (27%)
**Workshops**	1 (20%)	4 (40%)	13 (72%)	2 (50%)
< 4 h	1	2	1	1
5–9 h		1	4	
10–14 h		1	2	
> 15 h			6	1
**Conferences**	1 (20%)	5 (50%)	16	2 (50%)
< 4 h	1	2	1	1
5–9 h		2	1	
10–14 h		1	1	
> 15 h			13	1
**Other Training**	3 (60%)	6 (60%)	11 (%)	1 (25%)
< 4 h	1 ^*^	1		
5–9 h		2	1	
10–14 h				1
> 15 h		3	10	

^*^ Two respondents declined to quantify the hours they spent in other training

### Medical cannabis practice, knowledge, and comfort

**Fig. 2:**
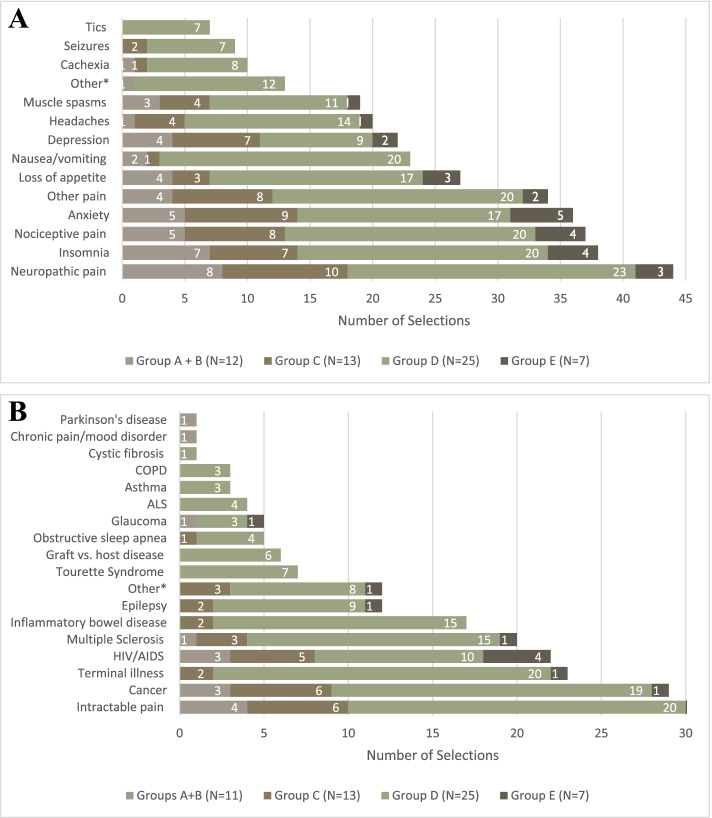
A. Do any of your patients use cannabis for medical purposes? If yes, for what symptoms do your patients use medical cannabis? (Select all that apply.). *Other = "movement disorders", "autism rage", "nightmares", "panic attacks", "drug sub", "anti-tumour", "diarrhea", "angioedema", "fatigue PTSD", "many conditions", "neuro symptoms", "PTSD", "medicalization". *N* = 1 each. 2B. Do any of your patients use cannabis for medical purposes? If yes, for what conditions do your patients use medical cannabis? (Select all that apply.). *Other = “dystonia”, “chronic pain”, “psoriasis”, “arthritis”, “fibromyalgia”, “angioedema”, “autism”, “drug addiction”, “drug dependence”, “menstrual pain”, unspecified. *N* = 1 each. COPD = Chronic Obstructive Pulmonary Disease. ALS = Amyotrophic Lateral Sclerosis. HIV/AIDS = Human Immunodeficiency Virus/Acquired Immunodeficiency Syndrome

Most (*n* = 40, 57%) respondents reported receiving more questions from patients regarding MC since recreational cannabis was legalized, but 19 (27%) reported being unfamiliar with the requirements for patients to obtain CMP in Canada. Most respondents (*n* = 57, 81%) had patients who use CMP. The most common symptoms for the use of CMP were pain (neuropathic and nociceptive) and insomnia (Fig. 2A), whereas the most common conditions for the use of CMP was intractable pain, followed by cancer (Fig. 2B). On average, only HCPs in Group D reported being comfortable or very comfortable with their knowledge of (4.56 ± 0.77 out of 5), counselling on (4.48 ± 0.96), or writing prescriptions for MC (4.44 ± 1.16).

### Group A + B – Respondents who do not recommend nor prescribe cannabis (*N* = 16, 23%)

For these HCPs, the strongest barrier for not recommending MC was the lack of sufficient scientific evidence to support its use for the indications for which their patients were seeking treatment (mean 3.13 ± 1.82 out of 5), followed by fear of interactions with other medications (2.31 ± 1.25) and reservations regarding signing a medical declaration to certify patients in a MC program (2.25 ± 1.77). Their weakest barriers were the Canadian Medical Association's position on cannabis for medical purposes (0.81 ± 1.17) and their health group or leadership not allowing or supporting medical cannabis (0.69 ± 1.30).

With regards prescribing MC, the largest barrier for these HCPs was not knowing which dose of cannabis to prescribe (mean 3.88 ± 1.36), followed by the lack of sufficient scientific information to support its use for the indications for which their patients were seeking treatment (mean 3.50 ± 1.71), as well as not having prescribing privileges (3.50 ± 2.00), and not knowing which cannabis formulation or method of administration to choose (both 3.19 ± 1.80). The weakest barriers were again, the Canadian Medical Association's position on CMP (0.88 ± 1.15) and their health group or leadership not allowing or supporting MC (0.75 ± 1.34).

Reasons for not referring a patient to a person/clinic with expertise in MC included not being familiar with the process for referral, not knowing who or where to refer patients, and the fact that patients did not request referrals (all *n* = 2).

### Group C – Respondents who recommend cannabis and refer patients to a person/clinic with expertise in medical cannabis for further evaluation, but do not prescribe it themselves (*n* = 14, 20%)

**Fig. 3:**
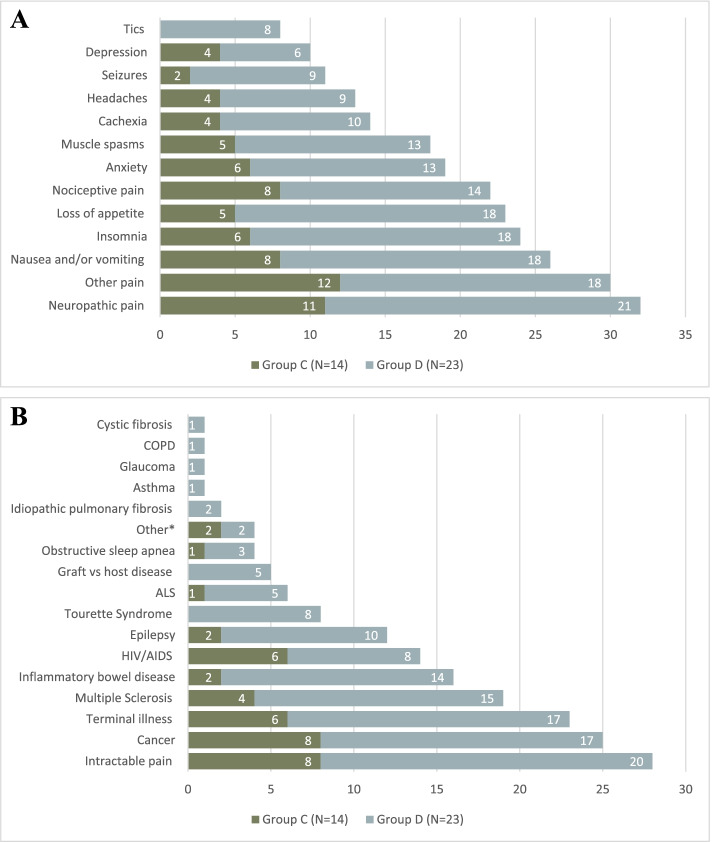
A. For what symptoms do you usually recommend medical cannabis? Select all that apply. 3B. For what conditions do you usually recommend medical cannabis? Select all that apply. *Other = "Neuropathy associated pain", "fibromyalgia", "itch", "variable reasons". COPD = Chronic Obstructive Pulmonary Disease. ALS = Amyotrophic Lateral Sclerosis. HIV/AIDS = Human Immunodeficiency Virus/Acquired Immunodeficiency Syndrome

HCPs in this category most often recommended MC for other pain (*n* = 12, 16% of their selections) and neuropathic pain (*n* = 11, 15%), followed by nausea/vomiting (*n* = 8, 11%) and nociceptive pain (*n* = 8, 11%) (Fig. 3A). They most often recommended MC to patients with cancer (*n* = 8, 20%) and intractable pain (n = 8, 20%), followed by terminal illness (*n* = 6, 15%), and Human Immunodeficiency Virus/Acquired Immunodeficiency Syndrome (HIV/AIDS) (*n* = 6, 15%) (Fig. 3B). They most often recommended MC in the form of oils (*n* = 10, 29% of selections) followed by capsules (*n* = 7, 20%) and edibles (*n* = 6, 17%). The largest barrier to prescribing MC for these HCPs was not knowing which dose of cannabis to prescribe (mean 3.50 ± 2.07 out of 5) and not having prescribing privileges (2.93 ± 1.86). The most common reason for referring a patient to a person/clinic with expertise in MC was not being comfortable prescribing cannabis and believing that a person/clinic with expertise in MC may be better equipped to prescribe MC (*n* = 11, 79% of selections).

### Group D – Respondents who recommend and prescribe cannabis if they think it is appropriate without referring to a person/clinic with expertise in medical cannabis (*n* = 25, 36%)

HCPs in this category most often recommended MC for neuropathic pain (*n* = 21, 12% of all selections), as well as other pain, nausea/vomiting, insomnia and loss of appetite (*n* = 18, 10% each) (Fig. 3A). They most often recommend MC for conditions involving intractable pain (*n* = 20, 15% of selections), cancer (*n* = 17, 13%) and terminal illness (*n* = 17, 13%) (Fig. 3B). Oils were the dosage form most often recommended (*n* = 22, 28% of selections) followed by capsules (*n* = 16, 20%) and vaporization (*n* = 15, 19%). The most common reasons for prescribing MC themselves, rather than referring patients to a person/clinic with expertise in MC, included feeling comfortable discussing the risks of adverse events (*n* = 21, 18% of selections) and evaluating the risk of drug interactions (*n* = 19, 17%), as well as knowing which cannabis formation to choose (i.e., THC vs CBD; *n* = 19, 17%) and which method of cannabis administration to recommend (*n* = 19, 17%). HCPs in this category did not necessarily only prescribe MC if symptoms are refractory to standard therapies (mean 3.04 ± 1.2). Most HCPs (*n* = 24, 96%) in this category assessed patients for the presence of potential contraindications or precautions to MC prescription, including anxiety and mood disorders (*n* = 21, 21% of selections) the risk of addiction (*n* = 21, 21%), cardiovascular disease risk factors (*n* = 20, 20%), and family history of psychosis (*n* = 20, 20%). Among various patient populations, they most commonly prescribed MC to patients taking high doses of opioids, benzodiazepines or other sedating mediations (*n* = 14, 39% of selections). About half of these participants asked patients to sign a written treatment agreement form and consent form (*n* = 13, 54% and *n* = 12, 50%, respectively).

### Opinions on cannabis for medical purposes

Of respondents, 11 (16%) chose not answer questions regarding whether or not they initiate conversations about MC or experience conversations initiated by their patients regarding MC. They were asked to share their opinions on various topics related to MC. They endorsed being likely to recommend MC most strongly if the results of clinical trials demonstrated safety and efficacy for the symptoms and conditions they were treating (3.36 ± 2.06). They were least likely to recommend MC on the basis of case reports demonstrating the same (1.91 ± 1.58). Almost all (*n* = 9; 81%) were uninterested or maybe interested in attending a workshop on MC; they most commonly preferred to learn about MC through clinical practice guidelines and webinars (both *n* = 6, 32% of selections). None of the respondents endorsed learning through a book (*n* = 0).

Among this group, the most commonly selected research priorities related to MC were long-term health effects (*n* = 10, 15%) and drug interactions (*n* = 8, 12%). Other commonly selected priorities were the effect of MC on children and adolescents; risk related to emphysema, bronchitis, or other lung disease; and effect of second-hand smoke/vapour (all *n* = 7, 11%).

### Recreational cannabis practice, knowledge, and comfort

The section of the survey relating to recreational cannabis was presented to all respondents. The majority reported asking their patients if they smoke, vaporize, or ingest cannabis for recreational purposes (83%, 67%, and 65% respectively). The majority (56%) also reported an increase in the frequency of questions regarding recreational cannabis since legalization. On average, the only respondents who reported feeling comfortable with their knowledge of, and counseling patients on, recreational cannabis were respondents in Group D (3.71 ± 1.40 and 3.54 ± 1.44, respectively).

### Impact of the COVID-19 pandemic on cannabis practice

**Fig. 4:**
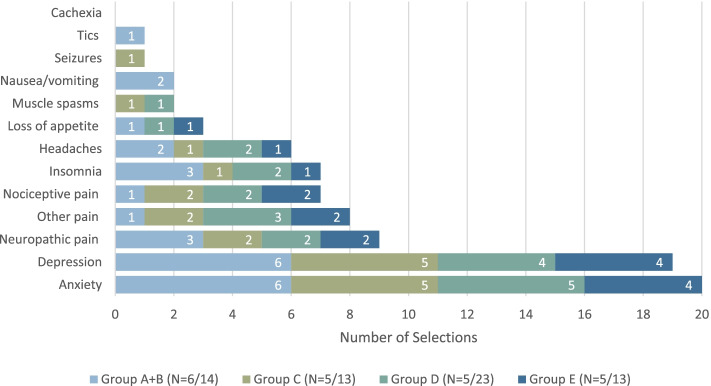
Have the reasons for cannabis use shifted during the pandemic? If yes, increased frequency of use for (select all that apply)

The section of the survey relating to the impact of the COVID-19 pandemic was presented to all respondents. The majority reported the frequency of questions they received (*n* = 39, 59%) as well as the prescriptions they provided for MC (*n* = 41, 68%) stayed the same during the pandemic, and this was reflected in all groups. Two thirds of respondents in groups A + B, C, and E, noted a greater frequency of overall cannabis use among their patients (*n* = 28, 67%); most respondents in Group D noted that changes in the patterns of cannabis use depended on the patient or stayed the same (*n* = 17, 74%). Across all groups, respondents who noted a shift in the reasons for cannabis use reported increased frequency of use for anxiety (*n* = 20) and depression (*n* = 19) (Fig. 4).

## Discussion

The legalization of recreational cannabis in Canada has sparked an increased interest is exploring the potential uses of cannabis and CBM to provide relief for various symptoms which may be refractory to first line treatments. Legalization has also contributed towards increased dialogue about a substance whose use still remains stigmatized. Our pan-Canadian survey of HCPs aimed to understand knowledge, comfort, and practice of cannabis for medical and recreational purposes, and respondents’ opinions on various topics related to CMP, as well as how the COVID-19 pandemic may have affected this aspect of respondents’ practices. This latter aspect is unique to our study.

Most of the respondents answering our survey were attending physicians, and a large proportion were from Ontario and Quebec. This may be because these two provinces have a higher concentration of HCPs compared to the rest of the country. For example, they make up 63% all physicians in Canada [14].

Over half of our overall sample already prescribe and/or recommend cannabis. However, as may be expected, those who are actually prescribing MC (Group D) felt, on average, comfortable with their level of knowledge related to MC. Of these respondents, most had sought out education about CBM outside of their medical training. Many HCPs in Canada may not be familiar with CBM due to the lack of coverage of this topic in professional school. Studies assessing medical education in Canada and around the world have found that most trainees have minimal exposure to CBM-related topics in their curricula [15–18]. With strong media and public interest surrounding CBM, HCPs are likely to be asked more questions about the topic. Therefore, even if they do not support or promote the use of CBM themselves, HCPs should be sufficiently knowledgeable to counsel patients. Going forward, advocacy is required to increase the coverage of CBM in the educational curriculum. Given the most commonly selected reasons for MC use in this survey (cancer or terminal-illness related symptoms and intractable pain) this may ideally take place during training in fields such as family medicine, palliative care, and pain management. This process could be facilitated by the creation of standardized teaching materials as well as the inclusion of cannabis-specific learning objectives in licensing exams and in the curricula of accreditation committees like the Committee on Accreditation of Canadian Medical Schools [15, 19]. Our results suggest that increased training, as in Group D, correlates with a stronger feeling of comfort in knowledge of, counselling on, and prescribing MC.

We originally conceived of this study prior to the COVID-19 pandemic and did not want to delay its execution given the uncertainty surrounding the pandemic. With increased numbers of people working from home, and with increased levels of isolation and mental health issues, we postulated that cannabis use may increase during the pandemic. Our hypothesis was supported by a study which reported increased MC use by cancer patients following the legalization of recreational cannabis in Canada [4]. In addition, lockdown measures aimed at limiting the number of infections and deaths from the COVID-19 was associated with increased cannabis use in the Netherlands between January 2019 and May 2020 [20]. Canadians who engaged in self-isolation during the pandemic were also found to have a 20% increase in cannabis use compared to those who did not[21], reflecting prior research that demonstrated increased cannabis use in times of economic recession [22]. Most respondents in our study reported no change in the frequency of prescriptions they made and questions they received regarding MC, and this makes sense, as most of the sample did not prescribe it themselves. However, the majority of respondents in Group A + B, C, and E (i.e., HCPs who do not prescribe it themselves and may therefore see a smaller proportion of their patients using cannabis regularly) noted a greater frequency of cannabis use among their patients during the pandemic. This was most commonly for anxiety and depression. This echoes research on the rise of anxiety and mood disorders as a consequence of the COVID-19 pandemic [23] and, indeed, preceding pandemics [24]. Importantly, however, the use of cannabis with comorbid mental illness is associated with an increased risk of adverse effects like psychosis and cannabis use disorder [25]. A recent scoping review demonstrated that feelings of boredom, depression, and anxiety during the pandemic contributed to increased cannabis use, which was associated with adverse clinical and psychiatric outcomes among users [26]. Furthermore, in another study, among participants who started using or increased their use of non-cannabis drugs during the pandemic, 40% reported doing so due to a change in access to MC [27].

Even outside the context of the pandemic, several respondents reported cannabis use among their patients, despite half the sample neither recommending nor prescribing MC. Furthermore, some reported being uncomfortable with the medico-legal aspects of MC access. The lack of HCP knowledge in this area could increase the risk of misuse, adverse effects, as well as poor clinical response in their patients. Altogether, these findings highlight the importance of i) more research and knowledge dissemination on safe and effective use of MC, particularly in depression and anxiety and ii) increasing HCP ability to counsel patients on appropriate use of MC, even if they do not recommend or prescribe it themselves.

The lack of scientific evidence to support the use of MC for certain indications was identified as a key barrier to recommending and prescribing MC. This echoes previous research [16] and may be overcome as more clinical trials are performed, and high-quality evidence accumulates. However, this may be hampered by the strict regulatory requirements for clinical trials involving CBM [28]. Furthermore, the private cannabis industry may be reluctant to invest in research due to the lack of intellectual property rights associated with naturally-occurring compounds [19]. Nevertheless, research in this area must be prioritized, as HCP uncertainty in the safety and efficacy of MC appears to be the strongest factor preventing more widespread adoption in clinical practice. By extension, results of clinical research should inform educational material on dosages, routes of administration, and proper prescription, as lack of such information was identified in our survey as a significant barrier to cannabis prescribing across all respondents. Clinical guidelines and webinars were the most popular methods of learning. This is possibly rooted in a preference for up-to-date, real-world evidence and clear guidance, i.e., training that is directly applicable to clinical practice. It is plausible that webinars were the most popular learning medium since the data collection period occurred during the pandemic, and this may have influenced the preference of learning.

Our study had important limitations which must be taken into consideration. First, we encountered challenges obtaining a sufficiently large sample to compare groups statistically. Although we requested for associations and colleges to forward our invite to members, some organizations cited specific policies regarding the distribution of surveys. For example, they would only distribute a survey if a member of their organization was involved or if it involved a school of pharmacy. Other organizations requested fees for survey distribution or denied the request on the basis that they receive too many survey invitations. While this survey was distributed to organizations across Canada, only 70 individuals completed the survey. Furthermore, as most respondents were from Ontario, Quebec, and Manitoba, results may not be representative of HCPs in other provinces and territories. Similarly, it is not clear whether this sample is representative of HCPs within each province. Therefore, results should be interpreted with caution as they may not be representative of the experiences and opinions of HCPs across Canada nor within particular provinces or territories.

In addition, it is plausible that members of an organization may respond to survey questions in a certain way, biasing results. For this reason, we provide a list of the organizations approached and those which agreed to distribute the survey. We also did not formally examine whether there was a relationship between years of training and participants’ responses, although it is possible that years in practice could potentially affect results. It is unclear exactly when MC training officially began in Canada and there remains a lack of standardized MC training among Canadian professional schools. Despite 20 years since the legal recognition of patients’ right to access MC, studies have consistently noted gaps in cannabis knowledge among trainees and professionals in medicine [17, 18], nursing [29], and pharmacy [30]. In one notable attempt at increasing knowledge, the Canadian Consortium for the Investigation of Cannabinoids initiated a continuing education program consisting of 64 live events in 26 locations in 2009–2010 [31]. Thus, it is appropriate to consider historical aspects of MC training when interpreting results related to educational needs. Furthermore, it is possible that the response rate would have been higher had the survey not been conducted during the pandemic, due to increased demand for HCP working hours and subsequent burnout.

Finally, some elements of our survey design could have been optimized. For example, the ordering of some questions could have been modified to assist the flow of data collection.

In some questions, only the option of unidirectional change in use was provided (i.e., increase, no change), but it would have been better to provide the option for change in bilateral directions (i.e., increase, no change, or decrease). Future surveys of this nature may wish to focus on HCPs most likely to be asked questions related to MC, such as family physicians, to determine more specific barriers to particular groups of HCPs. Similarly, future surveys may wish to focus on HCPs already involved in recommending and prescribing MC, to better understand their specific continuing education needs. Another limitation is that we did not specifically ask respondents whether they had authorization to prescribe MC. In Canada, MC is authorized by a prescriber who provides a medical document allowing individuals to obtain from a licensed producer or apply to Health Canada to grow their own. Physicians who choose to prescribe cannabis for medical purposes must be authorized by their provincial regulatory body [32]. We did not specifically ask HCPs to indicate whether they were authorized to prescribe cannabis for medical purposes. Over half of our survey respondents already recommend and prescribe MC, and many respondents endorsed a high level of comfort with their knowledge related to it. Although we examined individuals’ comfort with their level of knowledge, we did not measure knowledge objectively. Kruger et al*.* recently developed an 18-item questionnaire designed to assess knowledge of MC in relation to the current scientific knowledge[33]. Overall cannabis knowledge was related to perceived knowledge, and perceived competence in identifying harmful and irresponsible use of cannabis medicinally, but not comfort integrating cannabis into patients’ treatment regimens [33]. Nevertheless, understanding subjective barriers to recommending and prescribing MC remains a priority in order to inform policies that improve access.

## Conclusions

The majority of HCPs received little, if any, formal training in cannabinoid-based medicine in medical school or residency. Over half of respondents reported receiving more questions regarding MC since the legalization of recreational cannabis, and nearly one-third were unfamiliar with the requirements for obtaining CMP in Canada. Respondents endorsed discomfort with their knowledge of MC despite over 80% having patients who use CMP. The strongest barrier to prescribing was uncertainty in the dosage and routes of administration needed for effective and safe prescription. The strongest barrier to recommending or prescribing MC was the lack of sufficient clinical research demonstrating safety and efficacy. Many respondents noticed a greater frequency of cannabis use among their patients during the pandemic, especially for anxiety and depression. These findings suggest that medical training programs must reassess their curricula to enable HCPs to gain the knowledge and comfort required to meet the evolving needs of patients.

## Supplementary Information

**Additional file 1: Supplemental Table 1.** List of organizations contacted and organizations that agreed to circulate the survey. 

**Additional file 2:** Survey.

## Data Availability

The datasets used and/or analysed during the current study are available from the corresponding author on reasonable request.

## Abbreviations

ACMPR: Access to Cannabis for Medical Purposes Regulations
ALS: Amyotrophic Lateral Sclerosis
CBM: Cannabinoid-based medicine
CMP: Cannabis for medication purposes
COPD: Chronic Obstructive Pulmonary Disease
HCP: Healthcare practitioner
HIV/AIDS: Human Immunodeficiency Virus/Acquired Immunodeficiency Syndrome
MC: Medical cannabis
MMPR: Marihuana for Medical Purposes Regulations

## Footnotes

1 “Authorized” and “prescribed” will be used interchangeably in this manuscript. When authorizing MC, it is mandatory for Canadian physicians to indicate a certain limit of MC that is equivalent to dry cannabis products, in grams per day. This authorization may also include limitations on tetrahydrocannabinol (THC) content in all forms of MC.
