# Supplementary material for: Healthcare practitioner perceptions on barriers impacting cannabis prescribing practices
Source: BMC Complement Med Ther. 2022 Sep 8;22:237. doi: 10.1186/s12906-022-03716-9 (PMC9453734; doi:10.1186/s12906-022-03716-9)
Supplement: Supplementary file 1 — Additional file 1: Supplemental Table 1. List of organizations contacted and organizations that agreed to circulate the survey. [file 12906_2022_3716_MOESM1_ESM.docx]

**Supplemental Table 1** – List of organizations contacted and organizations that agreed to circulate the survey

| **Organizations Contacted** |
| --- |
| Canadian Society of Internal Medicine |
| Association des spécialistes en médecine interne du Québec(ASMIQ) |
| Association of Medical Microbiology and Infectious Disease Canada |
| Canadian Academy of Child and Adolescent Psychiatry |
| Canadian Academy of Geriatric Psychiatry |
| Canadian Academy of Psychiatry and the Law |
| Canadian Academy of Sport and Exercise Medicine |
| Canadian Anesthesiologists' Society |
| Canadian Association for the Study of Liver |
| Canadian Neurological Sciences Federation (CNSF) |
| Canadian Neurological Society (CNS) |
| Canadian Society of Clinical Neurophysiologists (CSCN) |
| Canadian Association of Child Neurology (CACN) |
| Canadian Society of Neuroradiology (CSNR) |
| Canadian Stroke consortium (CSC) |
| Canadian Association of Emergency Physicians |
| Canadian Association of Gastroenterology |
| Canadian Association of General Surgeons |
| Canadian Association of Interventional Cardiology |
| Canadian Association of Interventional Radiology |
| Canadian Association of Medical Biochemists |
| Canadian Association of Medical Oncologists |
| Canadian Association of Neuropathologists |
| Canadian Association of Nuclear Medicine |
| Canadian Association of Paediatric Surgeons |
| Canadian Association of Pathologists |
| Canadian Association of Physical Medicine and Rehabilitation |
| Canadian Association of Radiation Oncology |
| Canadian Association of Radiologists |
| Canadian Association of Thoracic Surgeons |
| Canadian Blood and Marrow Transplant Group (now known as Cell Therapy Transplant Canada) |
| Canadian Cardiovascular Society |
| Canadian College of Medical Geneticists |
| Canadian Critical Care Society |
| Canadian Fertility and Andrology Society |
| Canadian Geriatrics Society |
| Canadian Heart Rhythm Society |
| Canadian Ophthalmological Society |
| Canadian Orthopaedic Association |
| Canadian Paediatric Society |
| Canadian Pain Society |
| Canadian Psychiatric Association |
| Canadian Rheumatology Association |
| Canadian Sleep Society |
| Canadian Society for Clinical Investigation |
| Canadian Society for Transfusion Medicine |
| Canadian Society for Vascular Surgery |
| Canadian Society of Addiction Medicine |
| Canadian Society of Allergy and Clinical Immunology |
| Canadian Society of Cardiac Surgeons |
| Canadian Society of Colon and Rectal Surgeons |
| Canadian Society of Endocrinology & Metabolism |
| Canadian Society of Nephrology |
| Canadian Society of Otolaryngology — Head & Neck Surgery |
| Canadian Society of Palliative Care Physicians |
| Canadian Society of Pharmacology and Therapeutics |
| Association of Faculties of Pharmacy of Canada (AFPC) |
| Canadian Academy of the History of Pharmacy |
| Canadian Association for Population Therapeutics (CAPT) |
| Canadian Council on Continuing Education in Pharmacy (CCCEP) |
| Canadian Foundation for Pharmacy (CFP) |
| Canadian Society for Pharmaceutical Sciences (CSPS) |
| Canadian Society of Hospital Pharmacists (CSHP) |
| National Association of Pharmacy Regulatory Authorities (NAPRA) |
| Neighbourhood Pharmacy Association of Canada |
| The Pharmacy Examining Board of Canada |
| Alberta Pharmacists' Association (RxA) |
| Association des pharmaciens des établissements de santé du Québec (APES) |
| Association québécoise des pharmaciens propriétaires (AQPP) |
| British Columbia Pharmacy Association (BCPhA) |
| Ontario Pharmacists Association (OPA) |
| Pharmacists' Association of Newfoundland and Labrador (PANL) |
| Canadian Association of Physician Assistants |
| Canadian Orthopedic Residents' Association |
| Resident Doctors of Canada |
| Fédération des médecins résidents du Québec |
| Association of Residents of McGill |
| Professional Association of Residents of Ontario |
| Maritime Resident Doctors |
| Professional Association of Residents of Newfoundland & Labrador |
| Resident Doctors of Saskatchewan |
| Professional Association of Resident Physicians of Alberta |
| Professional Association of Residents and Interns of Manitoba |
| Resident Doctors of BC |
| Univeristy of Toronto Department of Medicine |
| McMaster University Department of Medicine |
| University of Saskatchewan Department of Medicine |
| McGill University Health Centre |
| University of British Columbia Department of Medicine |
| University of Calgary Department of Medicine |
| Ottawa Hospital Research Institute |
| **Organizations that Agreed to Circulate** |
| Canadian Society of Hospital Pharmacists (CSHP) |
| Cell Therapy Transplant Canada |
| Canadian Society of Endocrinology and Metabolism |
| Saskatchewan Health Authority |
| Association des spécialistes en médecine interne du Québec |
| Pharmacists’ Association of Newfoundland and Labrador |
| Canadian Association of Gastroenterology |
| National Association of Pharmacy Regulatory Authorities |
| Department of Obstetrics and Gynecology, McMaster University |
| University of Toronto Department of Family and Community Medicine |
| University of Calgary Department of Anesthesiology, Perioperative, and Pain medicine |
| Division of Infectious Diseases, University of Saskatchewan |
| Division of Internal Medicine, University of Toronto |
| Division of Gastroenterology and Hepatology, McGill University Health Center |
| Division of Infectious Diseases, The Ottawa Hospital |
| Department of Epidemiology and Biostatistics, Schulich School of Medicine, Western University |
| Division of Experimental Medicine, McGill University |
| Division of Infectious Diseases, Mcgill University Health Center |
| Ottawa Hospital Research Institute |
| Division of Molecular Medicine, McMaster University Faculty of Health Sciences |
| Department of Medicine, University of Montreal |
| Institute of Health Policy, Management and Evaluation, University of Toronto |
| Department of infectious diseases, Sunnybrook Health Sciences Centre |
| Department of Midwifery, BC Women’s Hospital |
