# Supplementary material for: Healthcare practitioner perceptions on barriers impacting cannabis prescribing practices
Source: BMC Complement Med Ther. 2022 Sep 8;22:237. doi: 10.1186/s12906-022-03716-9 (PMC9453734; doi:10.1186/s12906-022-03716-9)
Supplement: Supplementary file 2 — Additional file 2: Survey. [file 12906_2022_3716_MOESM2_ESM.docx]

**Barriers to access for medical cannabis and impact of the COVID-19 pandemic: Canadian healthcare providers perspectives**

Instructions

We are interested in understanding your knowledge, comfort and practice of cannabis for medical and recreational purposes. We are also interested in your opinions on various topics related to cannabis for medical purposes, and how the COVID-19 pandemic may have impacted on this aspect of your practice.

Attending physicians, clinical/research fellows, resident physicians, nurse practitioners, registered nurses, pharmacists, pharmacy resident or fellow, and physician assistants may complete this survey. The survey will take 10-minutes to complete. By completing the survey, you are providing your consent to participate in the study.

There are no conflicts of interest to declare related to this study. The information you provide is for research purposes only. You may choose to withdraw consent at any time without having to provide a reason. There are no risks associated with participating in the study.

Funding: The study is financially supported by an Investigator-Initiated Study grant from Tilray (Nanaimo, BC, Canada). Tilray has no role in study design, data collection, data analysis or data interpretation.

# Part I: Physician Demographics

1. **What is your sex?**

male

female

transgender

other

prefer not to answer.

1. **What is your age?** _________ years
2. **Were you born in Canada?**

no

yes

prefer not to answer.

1. **Are you:**

attending physician

clinical or research fellow

resident physician

nurse practitioner

registered nurse

physician assistant

pharmacist

pharmacy resident or fellow

none of the above

4a. If none of the above, screen out.

1. **In which province/territory do you practice?**

Ontario

Quebec

Nova Scotia

New Brunswick

Manitoba

British Columbia

Prince Edward Island

Saskatchewan

Alberta

Newfoundland and Labrador

Northwest Territories

Yukon

Nunavut

1. **What is your practice type? Select all that apply.**

allergy and immunology

anesthesiology

cardiology

cardiovascular surgery

clinical laboratory sciences

dermatology

dietetics

emergency medicine

endocrinology

family medicine

forensic medicine

gastroenterology

general surgery

geriatrics

gynecology

hepatology

infectious disease

intensive care medicine

internal medicine

nephrology

neurology

neurosurgery

obstetrics and gynecology

oncology

ophthalmology

orthopedic surgery

oral and maxillofacial surgery

otorhinolaryngology

palliative care

pathology

pediatrics

pediatric surgery

physical medicine and rehabilitation

plastic surgery

podiatry

proctology

psychiatry

pulmonology

public Health

radiology

rheumatology

surgical oncology

thoracic surgery

transplant surgery

urgent Care Medicine

urology

vascular surgery

other (specify):____________________________________

1. **How many years have you been practicing?** _________ (years)
2. **Did you have any training in cannabinoid-based medicines in professional school (ie, medical, nursing or pharmacy school)?**

no

yes

If yes, quantify the number of hours:

≤4 hours

5-9 hours

10-14 hours

≥ 15 hours

1. **Did you have any training in cannabinoid-based medicines during your practical training (ie, residency training for doctors, pharmacy clerkship for pharmacists or practical nursing rotations for nurses?**

no

yes

If yes, quantify the number of hours:

≤4 hours

5-9 hours

10-14 hours

≥ 15 hours

1. **Do you have any training in cannabinoid-based medicines outside of what you learned in professional school or your practical training program?**

no

yes

If yes, where did you receive this training? Select all that apply:

Workshops

If selected, then quantify the number of hours:

≤4 hours

5-9 hours

10-14 hours

≥ 15 hours

Conferences

If selected, then quantify the number of hours:

≤4 hours

5-9 hours

10-14 hours

≥ 15 hours

Other (specify):_________________

If selected, then quantify the number of hours:

≤4 hours

5-9 hours

10-14 hours

≥ 15 hours

# Part II: Medical cannabis practice, knowledge and comfort

The following section will evaluate your knowledge, comfort and practice of **cannabis for medical purposes**. We will ask you questions about recreational cannabis in a different party of the survey.

1. **Did you recommend or prescribe medical cannabis under the Marihuana Medical Access Regulations (MMAR), the Marihuana for Medical Purposes Regulations (MMPR), or Access to Cannabis for Medical Purposes Regulations (ACMPR) before recreational cannabis was legalized?**

no

yes

1. **How have you found the frequency of questions about medical cannabis have changed since legalization?**

more questions

stayed the same

fewer questions

1. **In the past 3 months, how many of your patients have enquired about medical cannabis?**

0

1

2-5

6-10

>11

1. **Are you familiar with the requirements for a patient to be able to obtain cannabis for medical purposes in Canada?**

no

yes

1. **Are you familiar with the requirements for a physician to be able to certify a patient for medical purposes in Canada?**

no

yes

1. **How comfortable do you feel with your knowledge of cannabis for medical uses?**

0 not at all comfortable 1 2 3 4 5 very comfortable

1. **How comfortable do you feel counseling patients on medical cannabis use?**

0 not at all comfortable 1 2 3 4 5 very comfortable

1. **How comfortable do you feel with writing prescriptions for medical cannabis indicating dosage, frequency of use, and method of administration?**

0 not at all comfortable 1 2 3 4 5 very comfortable

1. **Has a patient ever asked you to prescribe medical cannabis?**

no

yes

If yes, how many times have you been asked to prescribe medical cannabis in the past year?

1 time

2-5 times

6-10 times

>11 times

1. **Do any of your patients use cannabis for medical purposes?**

no

yes

**10a. If yes, estimate the number of patients that use medical cannabis: ___**

**10b. If yes, for what symptoms do your patients use medical cannabis?**

anxiety

depression

neuropathic pain

nociceptive pain

other pain

seizures

headaches

muscle spasms

loss of appetite

cachexia

nausea and/or vomiting

insomnia

tics

I don’t know

other, please specify: ______________

**10c. If yes, for what conditions do your patients use medical cannabis?**

Cancer

Glaucoma

HIV/AIDS

Tourette Syndrome

Amyotrophic Lateral Sclerosis

Epilepsy

Multiple Sclerosis

Inflammatory bowel disease, including Crohn’s disease

Obstructive sleep apnea

Asthma

Chronic obstructive pulmonary disease

Cystic fibrosis

Idiopathic pulmonary fibrosis

Intractable pain

Graft versus host disease

Terminal illness with probably life expectance of less than one year

I don’t know

other, please specify: ______________

1. **Do your patients initiate discussions about medical cannabis with you?**

no

yes

**11a. If yes, which of the following topics do your patients discuss with you? Select all that apply.**

efficacy of medical cannabis for the indication that the patient is interested in

safety of medical cannabis

methods of cannabis administration

formulations of cannabis

identify cannabis stores/dispensaries from which medical cannabis can be purchased

resources for patients to learn more about medical cannabis

process of certifying patients in the medical cannabis program

other, please specify:_________________

1. **Do you initiate discussions about medical cannabis with your patients?**

no

yes

**12a. If yes, which of the following topics do you discuss with your patients? Select all that apply.**

efficacy of medical cannabis for the indication that the patient is interested in

safety of medical cannabis

methods of cannabis administration

formulations of cannabis

identify cannabis stores/dispensaries from which medical cannabis can be purchased

resources for patients to learn more about medical cannabis

process of certifying patients in the medical cannabis program

other, please specify:_________________

**🡪 If Yes to question 11 or 12 above, then participants will be required to complete question 13 and Questionnaire A, B, C OR D**

**🡪 If No to question 11 or 12 above, then participants will skip to Part IIA *****

1. **Which of the following best describes your current clinical practice related to medical cannabis?**

I do not recommend or prescribe medical cannabis, nor do I refer patients to a person/clinic with expertise in medical cannabis for further evaluation **(A)**

I do not recommend or prescribe medical cannabis, but I refer patients to a person/clinic with expertise in

medical cannabis for further evaluation **(B)**

I recommend medical cannabis and refer patients to a person/clinic with expertise in medical cannabis for

further evaluation as I do not prescribe it **(C)**

I recommend and prescribe medical cannabis if I think it is appropriate without referring patients to a

person/clinic with expertise in medical cannabis **(D)**

**Questionnaire A - If the answer to question 13 is (A)**

**Questionnaire B - If the answer to question 13 is (B)**

**Questionnaire C - If the answer to question 13 is (C)**

**Questionnaire D - If the answer to question 13 is (D)**

****Questionnaire A ****

1. **How much of a barrier do you consider each of the following reasons to recommending medical cannabis?**

|  | **0**  No barrier | **1** | **2** | **3** | **4** | **5**  Very large barrier |
| --- | --- | --- | --- | --- | --- | --- |
| fear of adverse events |  |  |  |  |  |  |
| fear of interactions with other medications |  |  |  |  |  |  |
| fear of addiction |  |  |  |  |  |  |
| fear of long-term health effects |  |  |  |  |  |  |
| there is insufficient scientific evidence to support the use of cannabis for the indications that my patients are seeking treatment for |  |  |  |  |  |  |
| I do not support the use of cannabis for medical purposes |  |  |  |  |  |  |
| the Canadian Medical Association’s position on cannabis for medical purposes |  |  |  |  |  |  |
| I have reservations about signing medical declaration on the application to certify patients in the medical cannabis program |  |  |  |  |  |  |
| I have a suspicion that patients want to use cannabis for recreational purposes, and not medical purposes |  |  |  |  |  |  |
| my health group/leadership does not allow/support medical cannabis |  |  |  |  |  |  |
| other, please specify: ______________ |  |  |  |  |  |  |

1. **How much of a barrier do you consider each of the following reasons to prescribing medical cannabis?**

|  | **0**  No barrier | **1** | **2** | **3** | **4** | **5**  Very large barrier |
| --- | --- | --- | --- | --- | --- | --- |
| I do not have prescribing privileges |  |  |  |  |  |  |
| I do not know which method of cannabis administration to choose (e.g., capsules vs. oils) |  |  |  |  |  |  |
| I do not know which cannabis formulation to choose (e.g., THC vs. CBD) |  |  |  |  |  |  |
| I do not know which dose of cannabis to prescribe |  |  |  |  |  |  |
| I do not know where patients can obtain medical cannabis |  |  |  |  |  |  |
| I do not know how to certify patients in the medical cannabis program |  |  |  |  |  |  |
| fear of adverse events |  |  |  |  |  |  |
| fear of interactions with other medications |  |  |  |  |  |  |
| fear of addiction |  |  |  |  |  |  |
| fear of long-term health effects |  |  |  |  |  |  |
| there is insufficient scientific evidence to support the use of cannabis for the indications that my patients are seeking treatment for |  |  |  |  |  |  |
| I do not support the use of cannabis for medical purposes |  |  |  |  |  |  |
| the Canadian Medical Association’s position on cannabis for medical purposes |  |  |  |  |  |  |
| I have reservations about signing medical declaration on the application to certify patients in the medical cannabis program |  |  |  |  |  |  |
| I have a suspicion that patients want to use cannabis for recreational purposes, and not medical purposes |  |  |  |  |  |  |
| I don’t want to be identified as someone who prescribed medical cannabis |  |  |  |  |  |  |
| my health group/leadership does not allow/support medical cannabis |  |  |  |  |  |  |
| other, please specify: ______________ |  |  |  |  |  |  |

1. **Why do you not refer patients to a person/clinic with expertise in medical cannabis? Select all that apply.**

I do not support the use of cannabis for medical purposes

my health group/leadership does not allow/support medical cannabis

I did not know these services were available

I do not know which person/clinic to refer patients to

I am not familiar with the referral process for medical cannabis

my patients do not ask for referrals to a medical cannabis specialist

****Questionnaire B****

1. **How much of a barrier do you consider each of the following reasons to recommending medical cannabis?**

|  | **0**  No barrier | **1** | **2** | **3** | **4** | **5**  Very large barrier |
| --- | --- | --- | --- | --- | --- | --- |
| fear of adverse events |  |  |  |  |  |  |
| fear of interactions with other medications |  |  |  |  |  |  |
| fear of addiction |  |  |  |  |  |  |
| fear of long-term health effects |  |  |  |  |  |  |
| there is insufficient scientific evidence to support the use of cannabis for the indications that my patients are seeking treatment for |  |  |  |  |  |  |
| I do not support the use of cannabis for medical purposes |  |  |  |  |  |  |
| the Canadian Medical Association’s position on cannabis for medical purposes |  |  |  |  |  |  |
| I have reservations about signing medical declaration on the application to certify patients in the medical cannabis program |  |  |  |  |  |  |
| I have a suspicion that patients want to use cannabis for recreational purposes, and not medical purposes |  |  |  |  |  |  |
| my health group/leadership does not allow/support medical cannabis |  |  |  |  |  |  |
| other, please specify: ______________ |  |  |  |  |  |  |

1. **How much of a barrier do you consider each of the following reasons to prescribing medical cannabis?**

|  | **0**  No barrier | **1** | **2** | **3** | **4** | **5**  Very large barrier |
| --- | --- | --- | --- | --- | --- | --- |
| I do not have prescribing privileges |  |  |  |  |  |  |
| I do not know which method of cannabis administration to choose (e.g., capsules vs. oils) |  |  |  |  |  |  |
| I do not know which cannabis formulation to choose (e.g., THC vs. CBD) |  |  |  |  |  |  |
| I do not know which dose of cannabis to prescribe |  |  |  |  |  |  |
| I do not know where patients can obtain medical cannabis |  |  |  |  |  |  |
| I do not know how to certify patients in the medical cannabis program |  |  |  |  |  |  |
| fear of adverse events |  |  |  |  |  |  |
| fear of interactions with other medications |  |  |  |  |  |  |
| fear of addiction |  |  |  |  |  |  |
| fear of long-term health effects |  |  |  |  |  |  |
| there is insufficient scientific evidence to support the use of cannabis for the indications that my patients are seeking treatment for |  |  |  |  |  |  |
| I do not support the use of cannabis for medical purposes |  |  |  |  |  |  |
| the Canadian Medical Association’s position on cannabis for medical purposes |  |  |  |  |  |  |
| I have reservations about signing medical declaration on the application to certify patients in the medical cannabis program |  |  |  |  |  |  |
| I have a suspicion that patients want to use cannabis for recreational purposes, and not medical purposes |  |  |  |  |  |  |
| I don’t want to be identified as someone who prescribed medical cannabis |  |  |  |  |  |  |
| my health group/leadership does not allow/support medical cannabis |  |  |  |  |  |  |
| other, please specify: ______________ |  |  |  |  |  |  |

1. **Why do you refer patients to a person/clinic with expertise in medical cannabis for further investigation?**

patient requests a referral

I’m not comfortable recommending medical cannabis, and I believe a person/clinic with expertise in medical cannabis may be better equipped to assess patients for medical cannabis

I’m not comfortable prescribing medical cannabis, and I believe a person/clinic with expertise in medical cannabis may be better equipped to prescribe medical cannabis

I have reservations about signing medical declaration on the application to certify patients in the medical cannabis program

1. **To which person/clinic with expertise in medical cannabis do you refer your patients to? Select all that apply.**

a medical cannabis clinic, where a physician trained in medical cannabis can evaluate patients for medical cannabis

a colleague with expertise in medical cannabis

medical cannabis advocacy group

family physician

other, please specify: ______________

****Questionnaire C****

1. **For what symptoms do you usually recommend medical cannabis? Select all that apply.**

anxiety

depression

neuropathic pain

nociceptive pain

other pain

seizures

headaches

muscle spasms

loss of appetite

cachexia

nausea and/or vomiting

insomnia

tics

other, please specify: ______________

1. **For what conditions do you usually recommend medical cannabis? Select all that apply.**

Cancer

Glaucoma

HIV/AIDS

Tourette Syndrome

Amyotrophic Lateral Sclerosis

Epilepsy

Multiple Sclerosis

Inflammatory bowel disease, including Crohn’s disease

Obstructive sleep apnea

Asthma

Chronic obstructive pulmonary disease

Cystic fibrosis

Idiopathic pulmonary fibrosis

Intractable pain

Graft versus host disease

Terminal illness with probably life expectance of less than one year

other, please specify: ______________

1. **When you recommend medical cannabis, which dosage form do you usually recommend? Select all that apply.**

smoked

vaporized

oils

edibles

capsules

for some patients, I recommend a combination of the above, please specify

smoked and vaporized

smoked and oils

smoked and edibles

smoked and capsules

vaporized and oils

vaporized and edibles

vaporized and capsules

oils and edibles

oils and capsules

edibles and capsules

I recommend more than two forms of cannabis

I recommend cannabis, but refer patients to a specialist to determine dosage

1. **How much of a barrier do you consider each of the following reasons to prescribing medical cannabis?**

|  | **0**  No barrier | **1** | **2** | **3** | **4** | **5**  Very large barrier |
| --- | --- | --- | --- | --- | --- | --- |
| I do not have prescribing privileges |  |  |  |  |  |  |
| I do not know which method of cannabis administration to choose (e.g., capsules vs. oils) |  |  |  |  |  |  |
| I do not know which cannabis formulation to choose (e.g., THC vs. CBD) |  |  |  |  |  |  |
| I do not know which dose of cannabis to prescribe |  |  |  |  |  |  |
| I do not know where patients can obtain medical cannabis |  |  |  |  |  |  |
| I do not know how to certify patients in the medical cannabis program |  |  |  |  |  |  |
| fear of adverse events |  |  |  |  |  |  |
| fear of interactions with other medications |  |  |  |  |  |  |
| fear of addiction |  |  |  |  |  |  |
| fear of long-term health effects |  |  |  |  |  |  |
| there is insufficient scientific evidence to support the use of cannabis for the indications that my patients are seeking treatment for |  |  |  |  |  |  |
| I do not support the use of cannabis for medical purposes |  |  |  |  |  |  |
| the Canadian Medical Association’s position on cannabis for medical purposes |  |  |  |  |  |  |
| I have reservations about signing medical declaration on the application to certify patients in the medical cannabis program |  |  |  |  |  |  |
| I have a suspicion that patients want to use cannabis for recreational purposes, and not medical purposes |  |  |  |  |  |  |
| I don’t want to be identified as someone who prescribed medical cannabis |  |  |  |  |  |  |
| my health group/leadership does not allow/support medical cannabis |  |  |  |  |  |  |
| other, please specify: ______________ |  |  |  |  |  |  |

1. **Why do you refer patients to a person/clinic with expertise in medical cannabis for further investigation?**

patient requests a referral

I’m not comfortable recommending medical cannabis, and I believe a person/clinic with expertise in medical cannabis may be better equipped to assess patients for medical cannabis

I’m not comfortable prescribing medical cannabis, and I believe a person/clinic with expertise in medical cannabis may be better equipped to prescribe medical cannabis

I have reservations about signing medical declaration on the application to certify patients in the medical cannabis program

1. **To which person/clinic with expertise in medical cannabis do you refer your patients to? Select all that apply.**

a medical cannabis clinic, where a physician trained in medical cannabis can evaluate patients for medical cannabis

a colleague with expertise in medical cannabis

medical cannabis advocacy group

family physician

other, please specify: ______________

****Questionnaire D****

1. **For what symptoms do you usually recommend medical cannabis? Select all that apply.**

anxiety

depression

neuropathic pain

nociceptive pain

other pain

seizures

headaches

muscle spasms

loss of appetite

cachexia

nausea and/or vomiting

insomnia

tics

other, please specify: ______________

1. **For what conditions do you usually recommend medical cannabis? Select all that apply.**

Cancer

Glaucoma

HIV/AIDS

Tourette Syndrome

Amyotrophic Lateral Sclerosis

Epilepsy

Multiple Sclerosis

Inflammatory bowel disease, including Crohn’s disease

Obstructive sleep apnea

Asthma

Chronic obstructive pulmonary disease

Cystic fibrosis

Idiopathic pulmonary fibrosis

Intractable pain

Graft versus host disease

Terminal illness with probably life expectance of less than one year

other, please specify: ______________

1. **For what symptoms do you usually prescribe medical cannabis? Select all that apply.**

anxiety

depression

neuropathic pain

nociceptive pain

other pain

seizures

headaches

muscle spasms

loss of appetite

cachexia

nausea and/or vomiting

insomnia

tics

other, please specify: ______________

1. **For what conditions do you usually prescribe medical cannabis? Select all that apply.**

Cancer

Glaucoma

HIV/AIDS

Tourette Syndrome

Amyotrophic Lateral Sclerosis

Epilepsy

Multiple Sclerosis

Inflammatory bowel disease, including Crohn’s disease

Obstructive sleep apnea

Asthma

Chronic obstructive pulmonary disease

Cystic fibrosis

Idiopathic pulmonary fibrosis

Intractable pain

Graft versus host disease

Terminal illness with probably life expectance of less than one year

other, please specify: ______________

1. **When you prescribe medical cannabis, which dosage form do you usually prescribe? Select all that apply.**

smoked

vaporized

oils

edibles

capsules

for some patients, I recommend a combination of the above, please specify:

smoked and vaporized

smoked and oils

smoked and edibles

smoked and capsules

vaporized and oils

vaporized and edibles

vaporized and capsules

oils and edibles

oils and capsules

edibles and capsules

I recommend more than two forms of cannabis

I recommend cannabis, but refer patients to a specialist to determine dosage

1. **Why do you prescribe medical cannabis and not refer patients to a person/clinic with expertise in medical cannabis? Select all that apply.**

I have expertise in medical cannabis

I know which method of cannabis administration to choose (e.g., capsules vs. oils)

I know which cannabis formulation to choose (e.g., THC vs. CBD)

I am comfortable discussing the risk of adverse events

I am comfortable evaluating the risk of interactions with other medications

I believe there is sufficient scientific evidence to support the use of cannabis for the indications that my patients are seeking treatment for

other, please specify: ______________

1. **How much do you agree with the following statement: “I only prescribe cannabis if symptoms are refractory to standard therapies”**

strongly agree

somewhat agree

neutral

somewhat disagree

strongly disagree

1. **Which of the following, if any, do you do before prescribing cannabis for medical purposes? Select all that apply.**

I assess the risk of addiction

I evaluate family history of psychosis

I assess the patient for anxiety and mood disorders

I assess the patient for risk factors for cardiovascular disease

I assess the patient’s alcohol use

1. **Which of the following populations, if any, do you prescribe cannabis to? Select all that apply.**

heavy users of alcohol

patients taking high doses of opioids or benzodiazepines or other sedating medications

pregnant women

breastfeeding women

patients under the age of 18

none of the above

1. **When you prescribe medical cannabis, do you ask patients to sign a written treatment agreement?**

no

yes

1. **When you prescribe medical cannabis, do you ask patients to sign a consent form?**

no

yes

# Part IIA: Medical cannabis – Opinions, training and education

The following section will evaluate your opinion on various topics related to **cannabis for medical purposes**. We will ask you questions about recreational cannabis in a different party of the survey.

1. **Please rate the following statement: “Medical cannabis should be an option available to patients”**

strongly agree

somewhat agree

neutral

somewhat disagree

strongly disagree

1. **Please rate the following statement: “There should be a separate stream for medical vs. recreational cannabis”**

strongly agree

somewhat agree

neutral

somewhat disagree

strongly disagree

1. **Please rate the following statement: “Medical cannabis should only be prescribed by specialists who have undergone specific training and credentialing”**

strongly agree

somewhat agree

neutral

somewhat disagree

strongly disagree

1. **How likely are you to recommend or prescribe medical cannabis if:**

4a. you had adequate training in cannabinoid-medicine

0 not likely at all 1 2 3 4 5 very likely

4b. you had adequate training in certifying patients in the medical cannabis program

0 not likely at all 1 2 3 4 5 very likely

4c. colleagues in your field were prescribing it

0 not likely at all 1 2 3 4 5 very likely

4d. a nurse or trained counselor on cannabinoids were available for clinic support

0 not likely at all 1 2 3 4 5 very likely

4e. all other medical alternatives for the condition were not effective

0 not likely at all 1 2 3 4 5 very likely

4f. the results of clinical trials demonstrated safety and efficacy for the symptoms and conditions you were treating

0 not likely at all 1 2 3 4 5 very likely

4g. observational studies demonstrated safety and efficacy for the symptoms and conditions you were treating

0 not likely at all 1 2 3 4 5 very likely

4h. case reports demonstrated safety and efficacy for the conditions you were treating

0 not likely at all 1 2 3 4 5 very likely

4i. clinical management guidelines indicated the use of cannabis for the symptoms and conditions you were treating

0 not likely at all 1 2 3 4 5 very likely

1. **What do you feel are the research priorities related to medical cannabis? Select all that apply.**

long-term health effects

pharmacokinetics of different dosage forms

drug interactions

effect of second hand smoke/vapour

risk related to lung cancer

risk related to emphysema, bronchitis or other lung disease

risk related to cardiovascular disease

effects during pregnancy

effect on children and adolescents

use of cannabis in harm reduction strategies

safety related to driving and operating machinery

other, please specify: _________________________

1. **Would you be interested in attending a workshop on medical cannabis?**

no

yes

maybe

1. **What methods would you prefer to learn about medical cannabis? Select all that apply.**

practical interactive workshop

didactic workshop

webinar

book

conference

journal articles

clinical practice guidelines

other, please specify: _____________

# Part III: Recreational cannabis practice, knowledge and comfort

The following section will evaluate your knowledge and comfort with **cannabis for recreational purposes**.

1. **How have you found the frequency of questions about medical cannabis have changed since legalization?**

more questions

stayed the same

fewer questions

1. **How comfortable do you feel with your knowledge of cannabis for recreational use?**

0 not at all comfortable 1 2 3 4 5 very comfortable

1. **How comfortable do you feel counseling patients on recreational cannabis use?**

0 not at all comfortable 1 2 3 4 5 very comfortable

1. **Do you ask your patients if (select all that apply):**

they smoke cannabis for recreational purposes

vaporize cannabis for recreational purposes

ingest cannabis (e.g., edibles, oils) for recreational purposes

# Part IV: Impact of the COVID-19 pandemic on cannabis practice

1. **How have you found the frequency of questions you receive about medical cannabis has changed during the pandemic?**

more questions

stayed the same

fewer questions

1. **Have the patterns of cannabis use changed during the pandemic?**

Yes, greater frequency of use

stayed the same

Yes, lower frequency of use

It depends on the patient (some use more cannabis, some use less cannabis)

1. **How have you found the frequency of prescriptions for medical cannabis you provide has changed during the pandemic?**

more prescriptions

stayed the same

fewer prescriptions

1. **Have the reasons for cannabis use shifted during the pandemic?**

No, the reasons have stayed the same

Yes, increased frequency of use for

anxiety

depression

neuropathic pain

nociceptive pain

other pain

seizures

headaches

muscle spasms

loss of appetite

cachexia

nausea and/or vomiting

insomnia

tics

I don’t know

other, please specify: ______________

1. **Are there any comments you would like to make regarding this topic or this survey?**

**Thank you for taking the time to complete this survey.**
